# Experimental Study on the Performance of Hydraulic Vibration Assisted Broaching (HVAB) Based on Piezoelectric Sensors

**DOI:** 10.3390/s18082417

**Published:** 2018-07-25

**Authors:** Zhen Meng, Jing Ni, Yu Shi, Chuan-Yu Wu, Xiang-Qi Liu

**Affiliations:** 1 School of Mechanical Engineering, Hangzhou Dianzi University, Hangzhou 310018, China; mengzhen@hdu.edu.cn (Z.M.); 17816124684@163.com (Y.S.); 2 School of Mechanical Engineering and Automation, Zhejiang Sci-Tech University, Hangzhou 310018, China; cywu@zstu.edu.cn; 3 Ocean Engineering of Research Center, Hangzhou Dianzi University, Hangzhou 310018, China; lxiangqi@hdu.edu.cn

**Keywords:** vibration-assisted broaching, dual-valve electro-hydraulic exciter, broaching force, piezoelectric sensor, surface quality

## Abstract

In order to improve the keyway broaching process and verify the feasibility of vibration-assisted broaching process, an experimental study on a novel hydraulic vibration assisted broaching (HVAB) system with double-valve electro-hydraulic exciter (DVEHE) is proposed in this paper. The performances of HVAB at different excitation frequencies were compared from three aspects: (a) the cutting force under the different vibration frequencies, (b) the surface roughness of the workpiece, and (c) the flank face wear of the tool. For precision on-line measurement of larger broaching forces, four piezoelectric sensors were fixed on the broaching machine. The experimental results show that HVAB can effectively improve the performance of the broaching process, approximately reduce the broaching force by as much as 9.7% compared to conventional broaching (CB) and improve the surface quality of workpiece. Some explanations are offered to support the observations.

## 1. Introduction

Keyway broaching is a metal cutting process that gradually removes material by pulling a multi-toothed tool through a work piece, which has a wide field of application in automotive and aeronautic industries [1,2]. Because of the broach performing a sequence of roughing, semi-finishing and finishing operations in one stroke, it has processed productivity and outstanding accuracy of the machined surface [3,4]. Nowadays, in order to reduce broach wear and prolong tool life, the improvement of broaching process is becoming a research hotpot [5].

In the last two decades, in order to reduce the wear of broach and increase its lifespan, improve the surface precision of the workpiece [6], researchers have put much effort into optimizing the parameters of broaching tools, such as the modification of geometric model for the broaching tools [7,8], optimization parameters of rake angle, clearance angle and rise per tooth (RPT) [3,9,10]. Klocke et al. [11] used carbon free cutting material MC 90 to replace the speed steel for broach tool, which processed a higher hot hardness and increased thermal conductivity. Ni et al. [5] proposed a surface textured broach to improve broaching performance.

Vibration-assisted machining (VAM) is an effective method to obtain better cutting performance [12,13,14]. In early studies, VAM was used for traditional macro-scale metal-cutting applications [15]. In recent years, VAM systems have been applied in turning [16,17,18,19], milling [20,21,22,23,24], drilling [25,26,27] or grinding [28,29,30,31,32,33] and so on. In such research, VAM technique has advantage of the reducing cutting force and prolonging tool lifespan [34,35,36,37]. However, there are few studies on vibration-assisted broaching. We have used mathematical model and verification experiments to investigate the dynamics of broaching force of HVAB [12]. But further studies of HVAB will be needed to show the relationship between vibration parameters and broaching performances.

Studies have shown that the measuring method of broaching force can measure the cutting force from the local deformation of a machined component through a strain gauge [38]. However, this method requires that the mechanical structure to which the strain gauge is attached be able to generate local deformation, which has limitations. It is also possible to use an oil pressure sensor to measure the broaching force in the broaching process [39], but the oil pressure sensor does not have a significant effect on the measurement of the broaching force under vibration. It is also possible to estimate the cutting force by establishing a cutting force model [40,41]. For the dynamic broaching force in the HVAB proposed in this paper, a piezoelectric sensor with good dynamic response and high sensitivity was used to measure broaching force [42,43,44,45,46].

In this paper, aiming at the improvement of the broaching performance and the feasibility of process, the HVAB experimental scheme has been proposed to study the excitation frequency on the broaching force, surface roughness of the workpiece and the flank face wear of the tool. And in order to on-line measure the broaching force accurately, the measurement scheme of multi piezoelectric sensors has been first applied in broaching machine. This paper is organized as follows: Section 2 introduces the kinematics of HVAB process. The main parameters affecting the performance of HVAB are amplitude *a*_m_ and frequency *f*. Section 3 introduces the setup of experimental system and parameters. Section 4 shows the experimental data of broaching force, surface integrity and tool wear. Comparison and analysis under different parameters are also proposed in this section. Section 5 concludes with a summary of the paper and a future outlook.

## 2. HVAB Principle and Kinematics

**Figure 1:**
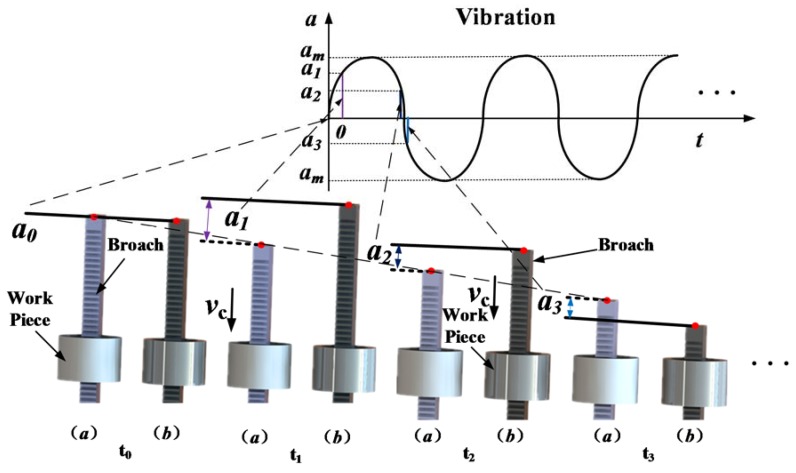
The process of (**a**) CB, and (**b**) HVAB.

Figure 1 schematically illustrates the principle of the VAB process. As shown in the figure, a simple harmonic motion (SHM) is generated in the broaching direction. Thus, compared to CB, VAB is an intermittent cutting process because of linear reciprocating motion during broaching.

According to Figure 1, the displacement and velocity of broach can be expressed as:

(1) $$x\left(t\right)=v_{c}t+a_{m}\sin(2\pi ft)\;$$

(2) $$v\left(t\right)=v_{c}+2\pi fa_{m}\cos(2\pi ft)\;$$

where, *x* denotes broach displacement, mm. *v* denotes broaching speed of HVAB, mm/s. *v*_c_ denotes broaching speed of CB, mm/s. *a*_m_ denotes displacement amplitude of SHM, mm. *f* denotes frequency of SHM, Hz.

Based on Figure 1, during CB process, the broach has been in contact with workpiece. But during HVAB process, the tool and workpiece are separated at *t*_0_. They are contact again at *t*_1_. And they are separated again at *t*_2_ (in one cycle). Therefore, the average broaching speed can be expressed as:

(3) $$v_{m}=v_{c}+\frac{2\pi fa_{m}\int_{t_{1}}^{t_{2}}\cos(2\pi ft)dt}{\Delta \tau }\;$$

According to Equations (1)–(3), during HVAB process, the actual removal length of the workpiece per cycle can be expressed as:

(4) $$\begin{array}{cc} l_{d} & =v_{c}(t_{2}-t_{1})+a_{m}[\sin(2\pi ft_{2})-\sin(2\pi ft_{1})]\\ & =v_{c}T+a_{m}[\sin(2\pi ft_{2})-\sin(2\pi ft_{0})]\\ & =\frac{v_{c}}{f} \end{array}\;$$

where, *l*_d_ denotes the relative displacement of broach and workpiece during each cycle, mm. *v*_m_ denotes VAB average broaching velocity, mm/s. *T* denotes the cycle of SHM, s.

## 3. HVAB Experiments

### 3.1. Experimental Setup

**Figure 2:**
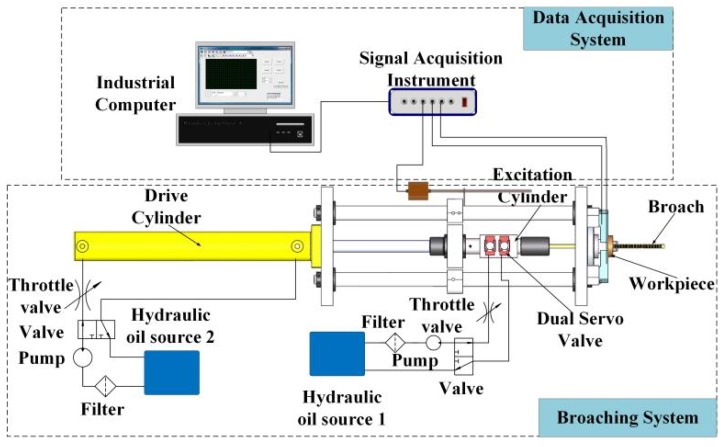
Schematic illustration of the HVAB test setup.

**Figure 3:**
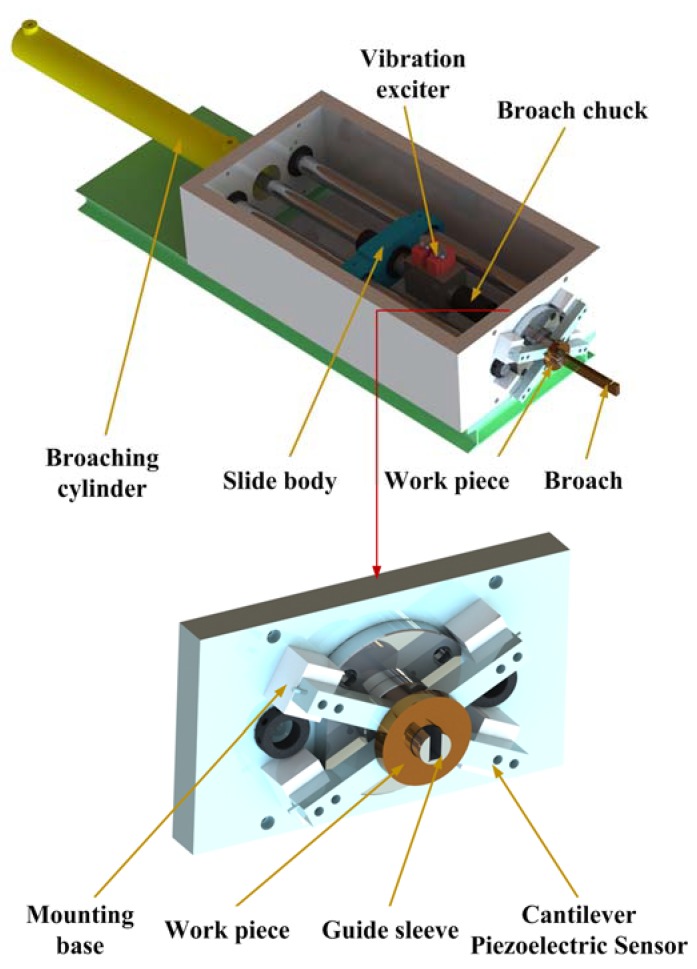
Schematic of the broaching system.

Figure 2 illustrates the schematic of the experimental setup. The whole experimental system can be broadly divided into two parts: broaching system and data acquisition system. Figure 3 shows the broaching system setup. Compared with a CB machine, a novel hydraulic exciter is installed on the broach machine, which generates the SHM on broach during cutting process. As shown in Figure 1, the exciter has changed the traditional broaching process, which converts hydraulic energy into mechanical energy used to drive the broach in cycle. The specific settings of the system are as follows: the back end of exciter is fixed on the slide body (coaxial with the broaching cylinder). And the broach chuck is fixed on the top end of exciter. The cylinder body is integrated with the valve seat. Otherwise, in order to on-line measurement of broaching force, the workpiece is fixed on four piezoelectric sensors, which are distributed around the guide sleeve and installed on the bed of broaching machine as shown in Figure 3.

**Figure 4:**
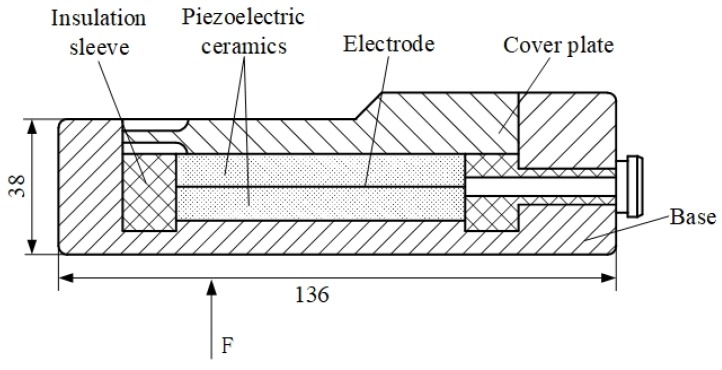
Structure diagram of piezoelectric sensor.

The actual setup has been carried out on LG61Ya horizontal broaching machine. The workpiece material is chosen as 1060 aluminum alloy and C45 steel. The keyway size is 40 × 16 × 1.7 mm after processing. The tool material used is M42. The broaching tool is shown in Figure 4, which is geometrically divided into roughing section (A), semi-finishing section (B) and finishing section (C). There are five new broaches with the same specification prepared for the tests. The experimental conditions and the geometric parameters of tool are listed in Table 1.

### 3.2. Measurement Method

In this paper, the tests are performed on two kinds of workpieces, and the frequencies varied as 0 Hz (CB), 10 Hz, 20 Hz, and 50 Hz by using the excitation signal cycle from control unit, whereas the broaching speed is kept at 40 mm/s. All the tests have been conducted at least three times to ensure repeatability of the results.

**Figure 5:**
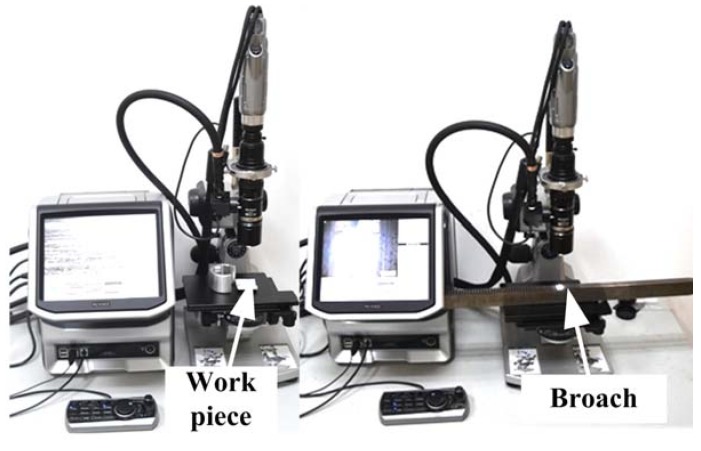
Work piece surface and broach wear observed.

In order to online measure broaching force, four piezoelectric sensors have been installed at the bottom of the workpiece as shown in Figure 3. The chosen piezoelectric sensor can produce small deformations under the broaching force. Then, the deformation of the piezoelectric film will be translated into a corresponding electrical signal in real time as shown in Figure 4. It is packaged in an alloy steel and has good pressure resistance [47]. The size is 136 × 32 × 38 mm. Piezoelectric sheets use piezoelectric ceramics, which have a strong piezoelectric effect and are widely used in sensors that measure dynamic response. It reflects the periodically changing cutting force; the natural frequency of the piezoelectric sheet is much higher than the natural frequency of the cutting force and is suitable for measuring the cutting force with large fluctuations in the exciting conditions [48,49]. And the piezoelectric film has fast response speed, high stability, which can measure the dynamic change of cutting force more accurately. Otherwise, the broaching stroke is obtained by a RHM0800S1DN05A01 (nonlinear ± 0.0015% FS, minimum ± 0.06 mm) giant magnetostrictive telescopic displacement sensor from the slide body, which is for positioning the broach. The above data were collected by the S7-300PLC CPU313C with four integrated AD channels. The signals from the sensor are sampled at the period of 2 ms. Borland C++ and Origin were used to present and graph the forces results. The surface quality and the tool wear are observed by a high speed microscope (KEYENCE VW-9000, KEYENCE, Osaka, Japan) as shown in Figure 5.

## 4. Results and Discussion

### 4.1. Comparison on Cutting Force

**Figure 6:**
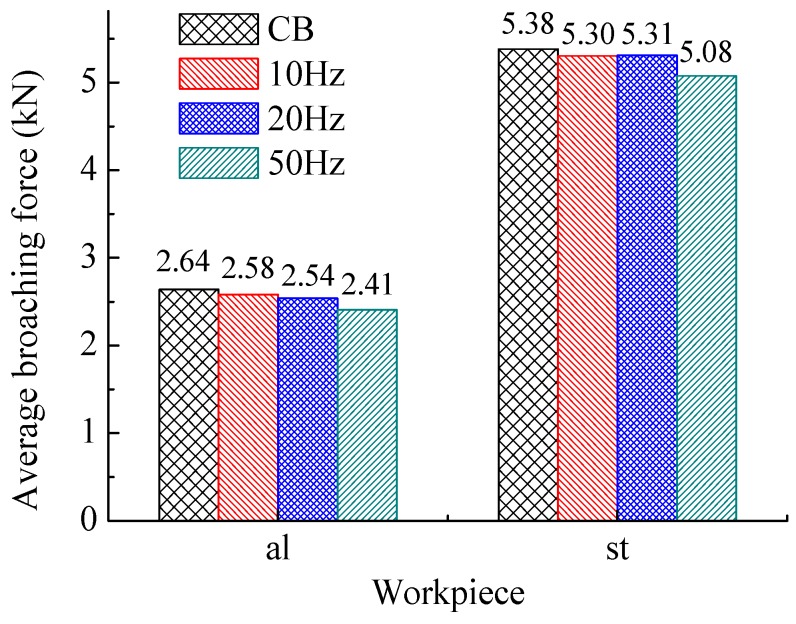
Average broaching forces of whole process.

**Figure 7:**
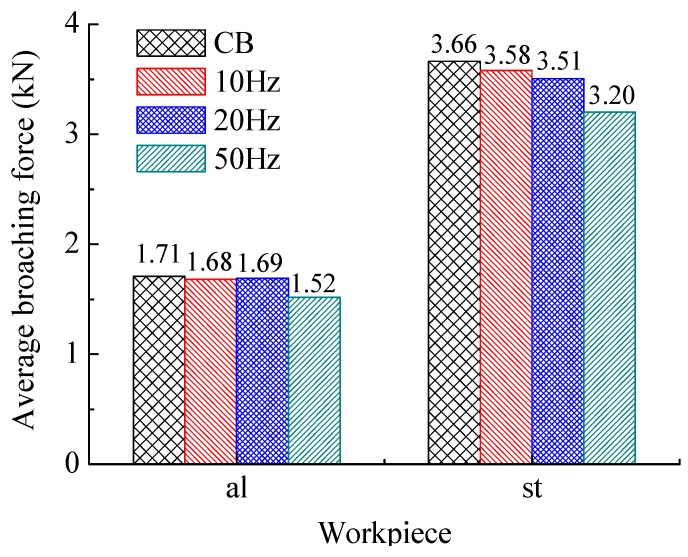
Average broaching forces of section A.

**Figure 8:**
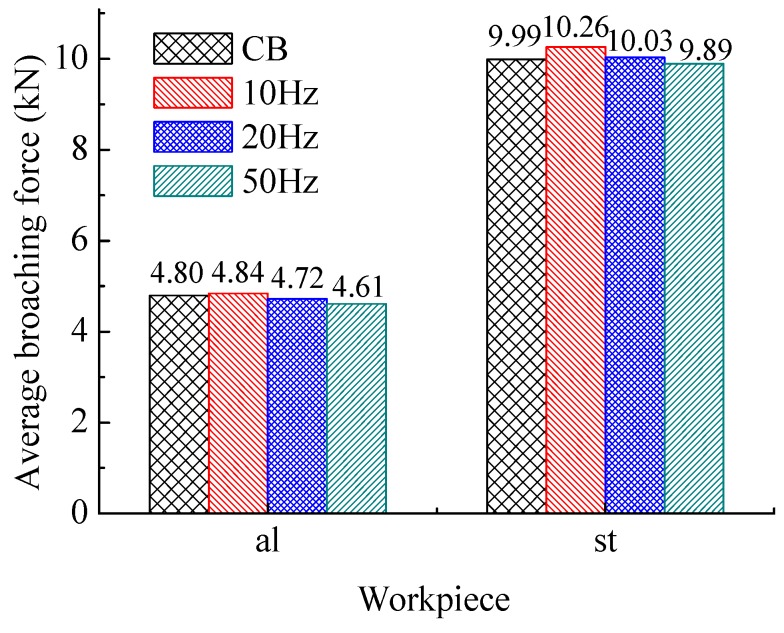
Average broaching forces of section B.

Figure 6 shows the comparison of the average of broaching forces in whole process. Figure 7 and Figure 8 show respectively the average values at different section. The ‘al’ means the work piece of 1060 aluminum alloy, and the ‘st’ means the one of C45 steel.

It can find that the broaching forces decrease with frequency increasing in general. The reason is that higher frequencies of vibration results in a smaller single of the amount volume of work piece in one time according to Equation (4). Thus, another explanation could be that the average effective cutting speed (corresponding only to the positive part of the translational speed; around 225 mm/s at 50 Hz for example) increases with the frequency increase; and the HVAB cutting force generally decreases with the cutting speed increase. Besides, the CB force can be expressed by [50]:

(5) $$F_{i}=K_{i}A_{i}\;$$

where, *A_i_* is the *i*th chip cross section area. *K_i_* is the specific cutting energy constant, which can be expressed by:

(6) $$\log K_{i}=a_{0}+a_{1}\log t_{c}\;$$

According to Equation (3), *t*_c_ has been reduced by SHM during broaching. Therefore, it is supposed to eventually lead to a smaller broaching force from Equations (5) and (6).

Figure 8 shows the broaching forces in the B section. The advantage of HVAB process is no more obvious on reduction broaching forces. Based on the previous studies of our team [51], it suggests that the incremental broaching force decrease the output amplitude of DVEHE. On other hand, the frequency of vibration should be higher to reduce the removal volume with incremental cutting area during broaching.

### 4.2. Comparison on Surface Integrity

**Figure 9:**
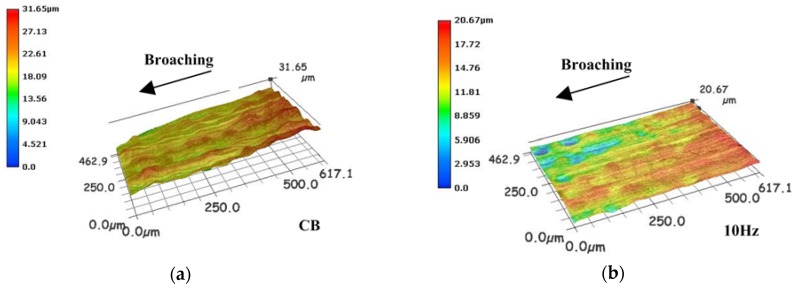

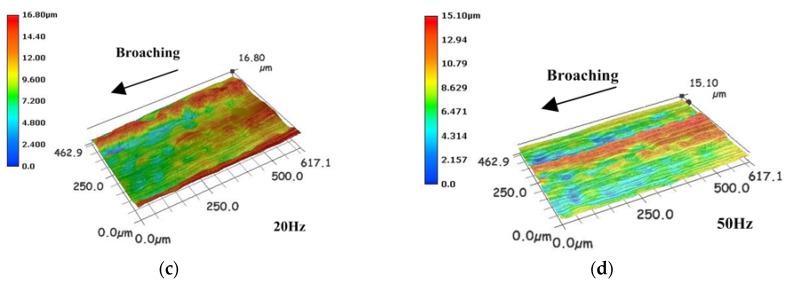
Workpiece surface (al) of (**a**) CB, (**b**) 10 Hz, (**c**) 20 Hz and (**d**) 50 Hz.

**Figure 10:**
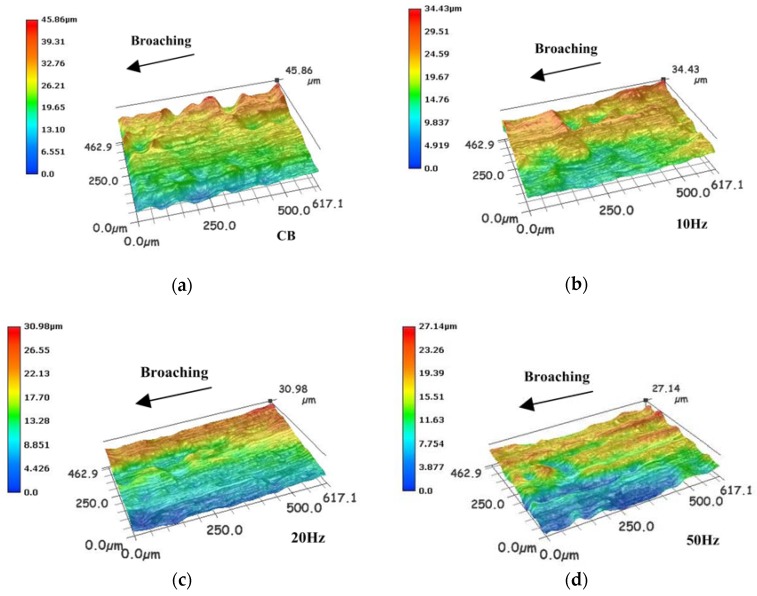
Workpiece surface (st) of (**a**) CB, (**b**) 10 Hz, (**c**) 20 Hz and (**d**) 50 Hz.

Figure 9 and Figure 10 show the measured results of the machined surface by the high speed microscope with the frequency of vibration varied as 0 Hz (CB), 10 Hz, 20 Hz, and 50 Hz. Without vibration in Figure 9a and Figure 10a, some scratches and irregularities appear on the machined surface.

Because of the characteristics of intermittent motion of broach, the HVAB technique can reduce the feed rate, and then improve the machined surface roughness compared with the CB. But broaching and feeding in the same direction, the reduction of feed rate is not obvious compared with other VAM. On the other hand, the higher vibration frequency results in a higher cutting energy to broaching material more effectively.

### 4.3. Comparison on Tool Wear

**Figure 11:**
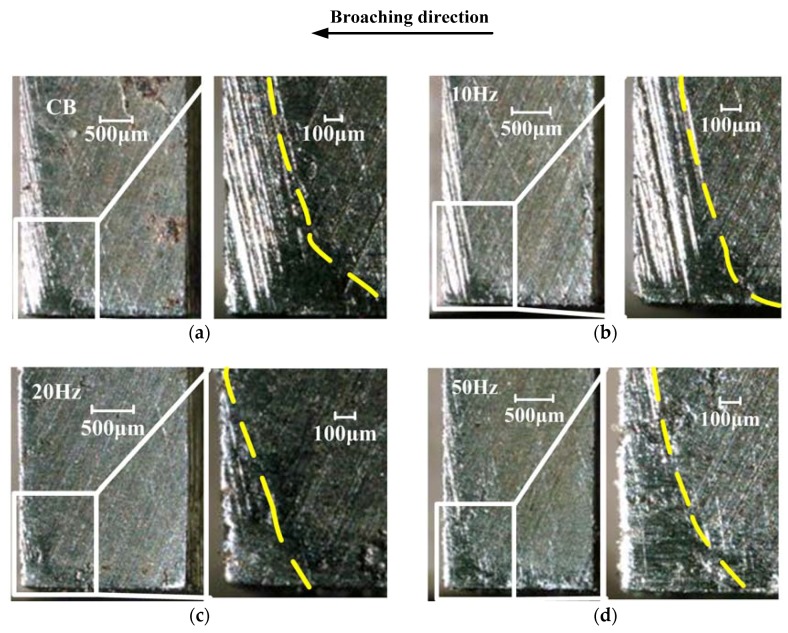
Tool wear of (**a**) CB, (**b**) 10 Hz, (**c**) 20 Hz and (**d**) 50 Hz.

The tool wear has strong influence on surface finish and also directly related to machining cost of the parts [15]. Figure 11 shows the photographs of flank faces of the thirtieth broach tooth with different vibration frequency. The wear of the thirtieth tooth could be the most serious, because the cutting area of thirtieth tooth is largest among all cutter teeth.

From the Figure 11, the maximum wear widths VBmax are 307.7 μm (CB), 235.8 μm (10 Hz), 132.1 μm (20 Hz) and 176.5 μm (50 Hz). It can be found that the area of wear at the flank face is significantly decreased, compared to CB. The reason is that the higher frequency induces comparatively shorter cutting time, which can lower broaching temperature on the cutting edge during broaching. But, compared Figure 11c,d, it shows that the wear width is bigger with frequency increase. The reason lies in that the higher vibration frequency induces comparatively longer tool-workpiece contact ratio, therefore the portion of each cycle where the broach is in contact with the workpiece is larger.

These results show that HVAB can reduce tool wear compared to CB. As the frequency increase, the tool wear gradually decrease. However, when the frequency exceeds a certain value, the wear of the tool will gradually increase.

## 5. Conclusions

In this paper, an experimental study on novel HVAB system for improving the keyway broaching performance and studying property of HVAB process. The influence of vibration-assisted broaching with different vibration frequencies on the cutting force, surface roughness and tool flank wear were studied and compared with traditional broaching process. In order to measure dynamic broaching force accurately, four piezoelectric sensors have been firstly installed on the broaching machine to realize the on-line detection. Based on the experimental results, the following conclusions can be compiled:

(1) HVAB can reduce broaching force by nearly 10% compared to CB, and in general the HVAB forces show more reduction as the frequency increases. The advantage of HVAB on reducing cutting forces is more obvious in Section A.
(2) HVAB is able to achieve better surface roughness at higher frequency. Because of the characteristics of intermittent broach motion, HVAB can reduce the feed rate, and thus improve the machined surface roughness compared with the CB. Therefore, the surface roughness of HVAB is mainly determined by the vibration the frequency and others.
(3) Higher frequency is useful for reducing the tool wear. Because the higher frequency can cause more cutting energy input and reduce the energy loss during the cutting process, it reduces the continuous engagement between the tool and the work piece, and both of them can maintain a lower temperature during the broaching process, but the higher frequency could induce longer tool-workpiece contact ratios.

This study shows that HVAB technique can apply in broaching process to improve broaching performance and increase broach lifespan.

## Figures and Tables

**Table 1:** Parameters of experimental system.

System Information		Value
Broaching machine	Driving mode	Hydraulic
	Supply pressure	10 MPa
	Broaching cylinder specification	80/50–500 mm
	Maximum broaching force	30 kN
	Broaching stroke	400 mm
	Hydraulic oil density	900 kg/m^3^
	Broaching velocity	35 mm/s
	Broaching cooling	Cooling liquid
Broach	Type	Keyway
	Size	595 × 16 × 40 mm
	Material	W18Cr4V
	Front height	34.52 mm
	Rear height	36.28 mm
	Tooth width	16 mm
	Rake angle of tooth	15 deg
	Clearance angle	3 deg
	Pitch	6 mm
	Number of tooth	45
DVEHE	Supply pressure	7 Mpa
	Maximum flow	100 L/min
	Excitation cylinder specification	80/50–10 mm
	Mass	12.6 kg
	Excitation valve	Rexroth 4WS2EM6
	Controller	S7-300PLC CPU314
	Frequency	0–100 Hz
	Amplitude	0–1 mm
